# The Feasibility of Maintaining Biological Phosphorus Removal in A-Stage via the Short Sludge Retention Time Approach: System Performance, Functional Genus Abundance, and Methanogenic Potential

**DOI:** 10.3390/ijerph19095494

**Published:** 2022-05-01

**Authors:** Haichao Luo, Wanqian Guo, Chuanming Xing, Bo Yan, Qi Zhao, Nanqi Ren

**Affiliations:** 1 State Key Laboratory of Urban Water Resource and Environment, Harbin Institute of Technology, Harbin 150090, China; lhcdxw_2010@163.com (H.L.); ab1529902467@163.com (C.X.); yanbo1968@163.com (B.Y.); zqhit@outlook.com (Q.Z.); 2 School of Environment, Harbin Institute of Technology, Harbin 150090, China; rnq@hit.edu.cn

**Keywords:** short sludge retention time, biological phosphorus removal, A-stage operational stability, functional microbial abundance, long-term methanogenic efficiency

## Abstract

The increasing concerns on resource and energy recovery call for the modification of the current wastewater treatment strategy. This study synthetically evaluates the feasibility of the short sludge retention time approach to improve the energy recovery potential, but keeping steady biological phosphorus removal and system stability simultaneously. SBR_S-SRT_ and SBR_control_ that simulated the short sludge retention time and conventional biological phosphorus removal processes, respectively, were set up to treat real domestic sewage for 120 d. SBR_S-SRT_ achieved an efficient COD (91.5 ± 3.5%), ${\mathrm{PO}}_{4}^{3-}$-P (95.4 ± 3.8%), and TP (93.5 ± 3.7%) removal and maintained the settling volume index around 50 mL/gSS when the sludge retention time was 3 d, indicating steady operational stability. The poor ammonia removal performance (15.7 ± 7.7%) and a few sequences detected in samples collected in SBR_S-SRT_ indicated the washout of nitrifiers. The dominant phosphorus accumulating organisms *Tetrasphaera* and *Hydrogenophaga*, which were enriched with the shortened sludge retention time, was in line with the excellent phosphorus performance of SBR_S-SRT_. The calculated methanogenic efficiency of SBR_S-SRT_ increased significantly, which was in line with the higher sludge yield. This study proved that the short sludge retention time is a promising and practical approach to integrate biological phosphorus removal in A-stage when re-engineering a biological nutrient removal process.

## 1. Introduction

The vast amounts of energy and material consumption caused by the huge scale and number of constructed wastewater treatment plants (WWTPs) has attracted society’s concerns. Thus, technologies that focused on energy saving or recovery [1,2], resource recovery or utilization [3,4], and meeting the strict effluent discharge standards [5] were developed. The certification of “energy neutral” was achieved in the Strass Wastewater Treatment plant that operated an A-B stage process, in Austria, Leading to the reconsideration of the A-B process to modify current WWTPs.

Generally, the first stage is the extremely high load biosorption of the biological concentration of sewage with minimum organic oxidation degradation. The concentrated sludge is anaerobically digested for the recovery of energy content from sewage organics. Subsequently, a low load biological stage follows the first stage, operated to ensure the removal of dissolved organics and ammonia. This is the so-called A-B process due to the two-stage operation model [6]. Thus, biological phosphorus removal is limited in a typical A-B process. Additionally, low available organic carbon in the B stage limits nitrogen removal.

To meet the strict discharge standards, enhanced biological phosphorus removal (EBPR) processes, such as anaerobic/anoxic–oxic (AO), anaerobic–anoxic–oxic (AAO), or their modified operation mode, are widely applied in WWTPs [7]. Generally, sequencing anaerobic and aerobic conditions is necessary to achieve efficient biological phosphorus removal [8]. However, the competition for insufficient organic carbon sources between polyphosphate accumulating organisms (PAOs) and denitrifying bacteria inevitably occurs in the anaerobic phase of WWTPs [9]. This unavoidable drawback would be overcome if biological phosphorus removal was separated from nitrogen removal when re-engineering a typical EBPR process. Based on the multiple autotrophic nitrogen removal technologies development and investigationin recent decades [10,11], modifying the classical A-B stage process into an A-stage for C and P removal, with the B-stage for autotrophic nitrogen removal, is a promising approach.

The high-rate activated sludge technology (sludge retention time, SRT 0.5–2 d) was employed as an A-stage for partial organic carbon capture, mainly based on bio-sorption in the typical A-B stage process [6]. However, the high-rate activated sludge technology lost sight of phosphorus removal, and chemical phosphorus removal was required to compensate for this deficiency. Integrating biological phosphorus removal into the A-stage might be an applicable and promising exploration when reengineering one EBPR process into the A-B stage. Furthermore, it would be really unfortunate if biological phosphorus removal was abandoned when reengineering one EBPR process into the A-B stage. Thus, researchers investigated the feasibility of efficient biological removal under short or ultra-short SRT [12,13]. Chan et al. operated sequencing batch reactors (SBRs) to treat synthetic sewage, reporting that phosphorus removal and system stability deteriorated when SRT was shorter than 3 d [14]. Shao et al. [15] explored the dynamics of organic carbon and phosphorus removal in an ultra-short SRT SBR system treating real domestic sewage. However, the sludge volume index (SVI) of the SBR system with SRT at 3 d was above 150 mL/g, indicating the system was at the edge of instability. The studies were mainly concerned with the removal of COD and ${\mathrm{PO}}_{4}^{3-}$-P. However, TP removal and the ammonia preservation in A-stage determine the necessity of a chemical phosphorus removal section and the applicability of autotrophic nitrogen removal, respectively. The attentions should be paid to the removal of total phosphorus (TP) and the preservation of ammonia when performance in real domestic sewage treatment. Additionally, the study evaluated the integration of short SRT EBPR in the A-stage, with sludge yield rate (*Y* or *Y_obs_*) or the ratio of the mixed liquor volatile suspended solids (MLVSS) and the mixed liquor suspended solids (MLSSs) [14,16]. Generally, waste activated sludge (WAS) anaerobic digestion (AD) is applied for energy recovery via biogas production in WWTPs [17], and the main objective of the A-B stage is energy and resource recovery. However, a few investigations evaluated the methanogenic efficiency of WAS from the S-SRT system by a long-term AD process.

The main objects of this study are to investigate the stability and the methane production potential of short-SRT EBPR systems acting as the A-stage. Two sequencing batch reactors (SBR_control_ and SBR_S-SRT_) are set up and operated for 120d. The oxic phase duration of SBR_control_ is adjusted (from 1.5 h to 2.5 h) to achieve efficient phosphorus and ammonia removal, while the SRT of SBR_S-SRT_ is adjusted from 5 d to 3 d. The overall COD, phosphorus removal efficiencies, and operational stabilities of the two SBR systems are studied; the microbial community and structures are measured to analyze the primary driving force corresponding to the reactors’ operations. Two AnSBR are also set up to anaerobically digest WAS from the two SBR systems treating sewage. This study proves the feasibility of short-time EBPR as a practical tool for the modification of conventional biological phosphorus removal processes into the A-stage towards improving the energy recovery potential.

## 2. Materials and Methods

### 2.1. Domestic Sewage and Reactors

**Figure 1:**
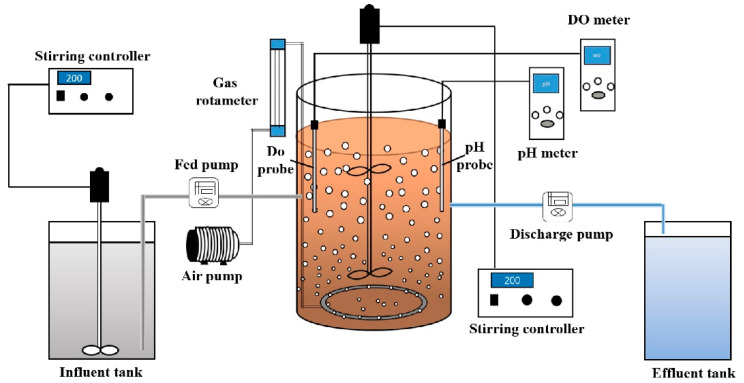
Schematic of the experimental SBR.

Domestic sewage was sampled from a sewer every day in the time period of 16:00–17:00. The sewage was collected from the second campus of the Harbin Institute of Technology and the residential quarters near the campus. A total of 40 L sampled sewage was passed through a 40 mesh sieve and stored in a 60 L influent tank. The main characteristics of the sewage sample are shown in Table 1. Two SBR reactors with a working volume of 9 L were set up in this study. The schematic diagram is shown in Figure 1. Two anaerobic SBR (AnSBR) were also set up to investigate the methanogen efficiency of WAS from the aforementioned SBRs. The working volume of the two AnSBRs were both 2 L.

### 2.2. Reactor Operation

Two SBR reactors were operated for four cycles per day. In each cycle, the incoming influent and discharge of effluent were both 4.5 L (half of the working volume) to maintain the hydraulic retention time as 12 h. The SRT of the SBR_control_ was 10 d by discharging 0.9 L mixture sludge at the end of the oxidation phase each day, but the discharging volume of the SBR_S-SRT_ reactors varied according to the pre-set SRT of each operation stage. Each cycle of SBR_S-SRT_ consisted of 15 min inflow at the first 15 min of the anaerobic phase, 2.5 h anaerobic, 1.5 h oxidation, 30 min settling, 15 min discharging, and 75 min idle. The SBR_control_ operated with the same durations of inflow, settling, discharging, and idle phase, but the duration of the anaerobic and oxidation phases varied. The biomass inoculum for the two experimental SBRs was collected from the second settling tank of the Wenchang Wastewater treatment plant (Harbin, China). The collected sludge was passed through 20 mesh sieves to remove large particles and then, the MLSSs were measured. Certain volumes of sludge were inoculated to maintain the initial MLSS concentrations of the two experimental SBRs as 4.0 g/L. Mechanical stirring was employed to maintain the suspension of the mixed liquor. Two air diffusers were set at the bottom of each SBR and connected with a glass rotameter to control the aeration intensity at 0.8 L/min. The oxygen concentration was measured daily. Additionally, the dissolved oxygen concentration of the two experimental SBRs was maintained above 3.0 mg/L. Each SBR was equipped with an air pump. All phases of each SBR were managed by time controllers. The detailed operational strategies of the two SBRs are shown in Table 2. The operation SRT and temperature of the two AnSBRs were 20 d and 37.0 °C, respectively. The detailed operation parameters of the two AnSBR are shown in the Supplementary Material Text S1 (Figure S1).

### 2.3. Analytical Methods

#### High-Throughput Sequencing Analysis

Sludge samples (100 mL) were collected from the two SBRs on the last day of each operation stage, and the inoculum WAS was sampled as a control. Metagenomic DNAs were extracted by using the 3S DNA isolation kit for Environmental Samples (Shanghai Majorbio Bioscience & Technology, Shanghai, China) following the manufacturer’s instructions. The primers 338F (ACTCCTACGGGAGGCAGCA) and 806R (GGACTACHVGGGTWTCTAAT) were employed for PCR performances, executed in TransStart Fastpfu DNA Polymerase 20 μL reaction systems through 27 time cycles. The high-throughput sequencing analysis of DNA samples was carried out by a commercial service and conducted based on the online data processing developed by the Shanghai Major Biomedical Science and Technology Ltd. (Shanghai, China) (http://www.majorbio.com/) on 1 December 2021.

COD, ${\mathrm{NH}}_{4}^{+}$-N, ${\mathrm{PO}}_{4}^{3-}$-P, total phosphorus (TP), MLSS, MLVSS, and SVI were measured daily, according to the standard methods (APHA 2012). The indexes of influent were measured without pre-filtration by 0.45 μm polyethersulfone filter. The generated biogas from the AnSBR was collected by a 2 L gas bag. The methane contents were measured by gas chromatography (Agilent 7890, Agilent Co., Ltd., City of Santa Clara, CA, USA) that was equipped with a flame ionization detector and a thermal conductivity detector.

As the energy or resource recovery is mainly based on the collected WAS, the accumulated WAS discharge per unit of *COD* (*Y_obs_*) was calculated by Equation (1) in this study.

(1) $$Y_{obs}=\frac{\sum (V_{discharge}\times S_{t})}{\sum V_{inf}\times \left(COD_{inf}-COD_{eff}\right)}$$

where *V_discharge_* (L) is the volume of discharged WAS; *S_t_* (g/L) is the concentration of MLSS or MLVSS in SBRs at time *t*; *V_inf_* (L) is the volume of influent sewage per day; *COD_inf_* (mg/L) and *COD_eff_* (mg/L) are the concentrations of *COD* in influents and effluents of SBRs.

The methanogenic efficiency (MP) of WAS and total methane recovery potential (MP_total_) of the two experimental SBRs were calculated according to Equations (2) and (3) as follows:

(2) $$\mathrm{MP}=\frac{V_{\mathrm{biogas}}\times R_{\mathrm{methane}}}{S_{\mathrm{WAS}}}$$

(3) $${\mathrm{MP}}_{\mathrm{total}}={\mathrm{MP}}_{\mathrm{average}}\times Y_{obs}\times R_{COD}$$

where V_biogas_ is the total volume of generated biogas at time t; R_methane_ is the volume ratio of methane in generated biogas; S_WAS_ is the concentration of inflow WAS corresponded to biogas production; and MP_average_ is the average methanogenic efficiency of specified operation duration. *Y_obs_* of the same specified operation duration was calculated according to Equation (1), and *R_COD_* is the average *COD* removal efficiency.

## 3. Results and Discussion

### 3.1. Operational Stability of Two SBRs under Different Stages

**Figure 2:**
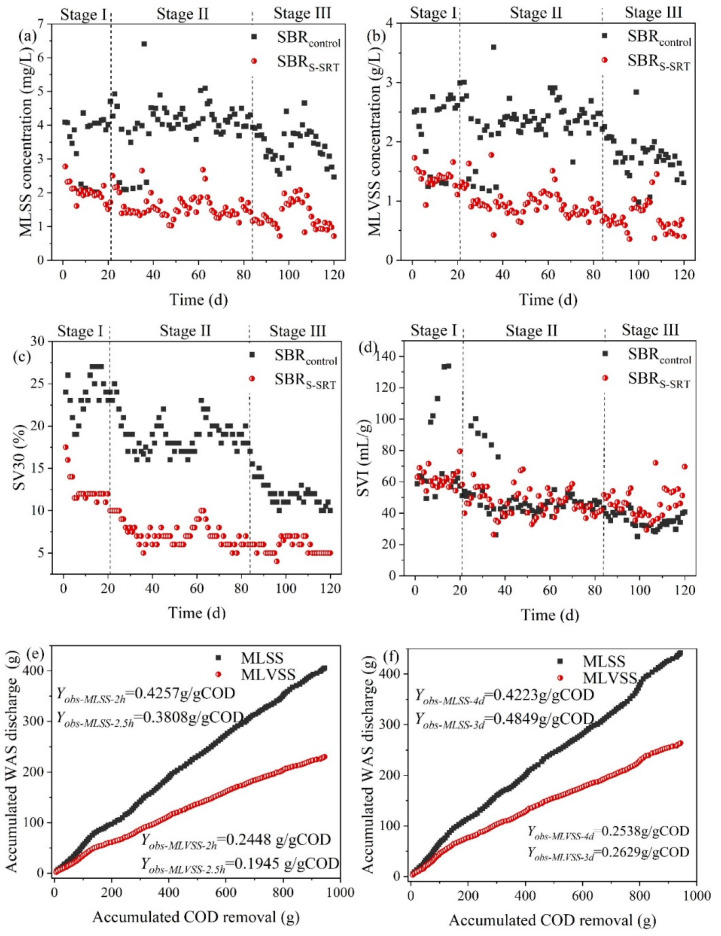
Variations of the sludge (**a**) MLSS and (**b**) MLVSS concentrations, (**c**) SV30 and (**d**) SVI in the two experimental SBRs and the accumulated WAS discharge of (**e**) SBR_control_ and (**f**) SBR_S-SRT_.

The operational stability was assessed by the measurements of MLSS, MLVSS, SVI, and *Y_obs_* as shown in Figure 2. The average MLSS, MLVSS, SVI, MLVSS/MLSS, and the *p*-value (*t*-test) of the two SBRs during each stage are shown in Table S1. The average concentrations of MLSS in SBR_S-SRT_ decreased with the decrease in SRT, as shown in Figure 2a. The average ratios of MLVSS/MLSS decreased from 60.2 ± 6.1% to 54.0 ± 8.0% with the SRT shortening from 4 d to 3 d in SBR_S-SRT_, which was not consistent with the study in [15]. As shown in Table S1, the average MLVSS/MLSS ratios of SBR_S-SRT_ remain slightly higher than SBR_control_ (54.0 ± 8.0% vs. 51.2 ± 6.3%, *p* = 0.10). This phenomenon might be caused by the difference and instability of sewage quality. However, a higher MLVSS/MLSS was beneficial to the sequential AD treatment of WAS [18].

The SV30 and SVI were measured to assess the sedimentation performance of the activated sludge in the experimental reactors and the results are shown in Figure 2c,d. The SV30 of SBR_control_ significantly decreased when the duration of the oxic phase was extended from 2 h to 2.5 h, while the SV30 remained relatively stable with the shortening of SRT in SBR_S-SRT_. The SVI of both experimental SBRs remained around 50 mL/g, indicating the excellent sedimentation capacity of the activated sludge in this study. It was notable that the SVI of SBR_S-SRT_ was higher than SBR_control_ during operation stage III. It might be due to the high organic substance content and also indicated the higher metabolic activity of the activated sludge in SBR_S-SRT_.

The accumulated WAS discharged from SBR_control_ and SBR_S-SRT_ is demonstrated in Figure 2e,f. The corresponding *Y_obs_* of MLSS and MLVSS were calculated by Equation (1), and the effluent MLSS or MLVSS was not included to represent the total feedstock of the subsequent WAS disposal processes realistically in this study. The extension of the oxidation duration slightly affected the *Y_obs_* of the SBR_control_, which were 0.4257 g/gCOD (*Y_obs-MLSS-_*_2.5*h*_) and 0.3808 g/gCOD (*Y_obs-MLSS-_*_2*h*_) when the oxic phase durations were 2 h and 2.5 h, respectively. The shortening of SRT from 4 d to 3 d increased the *Y_obs_* from 0.4223 g/gCOD (*Y_obs-MLSS-_*_4*d*_) to 0.4849 g/gCOD (*Y_obs-MLSS-_*_3*d*_). The *Y_obs-MLSS-_*_3*d*_ of SBR_S-SRT_ was 27.3% higher than the *Y_obs-MLSS-_*_2*h*_ of the SBR_control_ and the *Y_obs-MLVSS-_*_3*d*_ was 35.2% higher than *Y_obs-MLVSS-_*_2.5*h*_, indicating that more removed COD was converted into WAS. The high accumulation of WAS production of SBR_S-SRT_ at SRT 3 d indicates the outstanding COD capture capacity of SBR_S-SRT_. It was beneficial to increase the energy recovery potential of the subsequent WAS AD process.

### 3.2. Sewage Nutrient Removal Efficiencies of the Two SBR Systems

**Figure 3:**
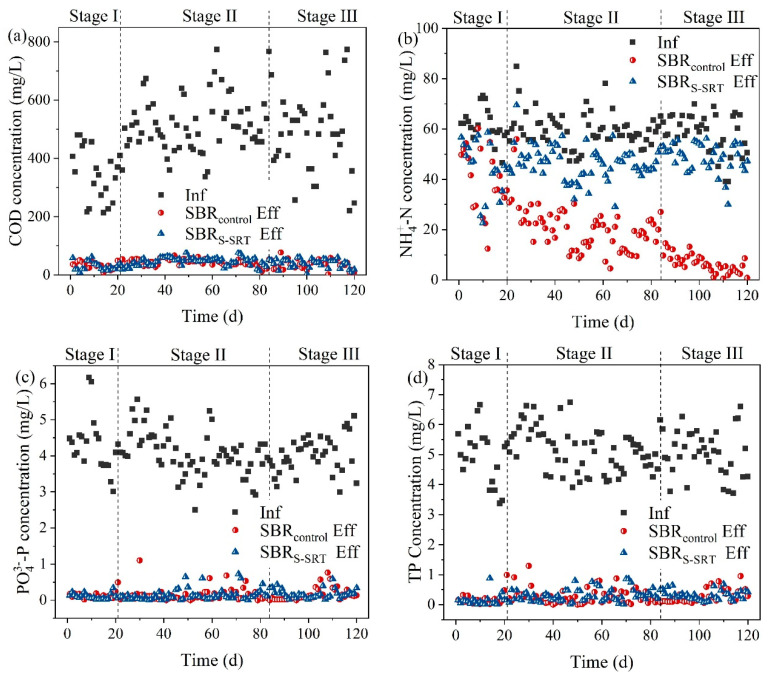
Performances of the two experimental SBRs on (**a**) COD, (**b**) ${\mathrm{NH}}_{4}^{+}$-N, (**c**) ${\mathrm{PO}}_{4}^{3-}$ -P, and (**d**) TP removal.

The SRT of the SBR_S-SRT_ and SBR_control_ was 5 d and 10 d, respectively, during the initial 21 d to accumulate PAOs and to acclimate to the operation mode for the start-up. The nutrient removal efficiencies of SBR_S-SRT_ and SBR_control_ EBPR reactors were shown in Figure 3. The average nutrient removal efficiencies were shown in Table S2. The average COD removal efficiencies of SBR_S-SRT_ and SBRcontrol under each stage were 90.0 ± 3.9% and 90.7 ± 5.4%, 91.1 ± 3.2% and 90.7 ± 3.5%, 92.0 ± 3.8% and 91.5 ± 3.5%, respectively (*p* > 0.05), as shown in Table S2. The shortening of the SRT affected the COD removal performance of SBR_S-SRT_ negligibly, while the extension of the oxic phase duration slightly enhanced the COD removal efficiency of SBR_control_, as shown in Figure 3a. COD was mainly removed by the adsorption of sludge flocs and the metabolism of activated organisms. The previous studies confirmed that short SRT enhanced the adsorption of sludge flocs and accelerated the microorganism activities [15], which accounted for the steady and excellent COD removal efficiency of SBR_S-SRT_.

Ammonia removal was the premise step that supplied ${NO}_{x}^{-}$-N for denitrification to achieve nitrogen removal in conventional WWTPs. The oxic phase duration of the SBR_control_ was adjusted and extended to achieve efficient ammonia removal. The ammonia removal performances of the two experimental SBRs are shown in Figure 3b. The ammonia removal efficiency increased from 35.2 ± 19.9% to 88.9 ± 5.4% with the increase in the oxic phase duration from 1.5 h to 2.5 h, as shown in Table S2. However, the ammonia removal efficiencies decreased from 25.9 ± 18.5% to 15.7 ± 7.7% (*p* < 0.05, Table S2) with the SRT shortening from 5 d to 3 d indicating that only a limited amount of ${\mathrm{NH}}_{4}^{+}$-N was removed in SBR_S-SRT_. An SRT around 4.3 d and a low dissolved oxygen were beneficial for the nitrification and the accumulation of AOBs [19]. The short duration of the oxic phase in SBR_S-SRT_ accounted for the inefficient ammonia removal. The activity of the AOBs was inhibited when the SRT of the SBR system was 3.5 d [13]. The nitrification was even undetectable in a continuous A/O process when the SRT was 4 d [17]. However, the SBR_S-SRT_ offered a lower ammonia removal efficiency, which was mainly by microbial assimilation.

Both the SBR_control_ and the SBR_S-SRT_ removed ${\mathrm{PO}}_{4}^{3-}$-P efficiently, as shown in Figure 3c. The average ${\mathrm{PO}}_{4}^{3-}$-P removal efficiency of SBR_S-SRT_ was above 95% under both SRT 4 d and 3 d, as shown in Table S2. It was comparable with the S-SRT or traditional EBPR processes of previous studies [17,20]. However, the evident value difference between the ${\mathrm{PO}}_{4}^{3-}$-P and TP content in sewage indicated the necessity to pay attention to the TP removal performance of the SBR_S-SRT_ and the SBR_control_. Furthermore, TP was the control target specified in effluent discharge standards. The average TP removal efficiencies of the SBR_S-SRT_ were 93.8 ± 3.9% and 93.5 ± 3.47% at SRT 4 d and 3 d, respectively, as shown in Figure 3d. The corresponding TP concentrations in the effluent were 0.32 ± 0.21 mg/L and 0.32 ± 0.16 mg/L, respectively, which met the first A class of Chinese standards (GB, 18918-2002, <0.5 mg/L). The TP in sewage consisted of both inorganic phosphorus (mainly ${\mathrm{PO}}_{4}^{3-}$-P) and organic phosphorus. Organic phosphorus was removed by biodegradation and biosorption. The different removal pathways of organic phosphorus and ${\mathrm{PO}}_{4}^{3-}$-P might account for the lower TP removal efficiency than that of ${\mathrm{PO}}_{4}^{3-}$-P. However, the chemically enhanced phosphorus removal section was non-essential in the subsequent B-stage. In conclusion, the low COD concentrations, high ammonia retained ratios, and excellent phosphorus removal confirmed that the effluent from the SBR_S-SRT_ was adapted to the autotrophic nitrogen removal processes and the additional chemical phosphorus removal section was non-essential.

### 3.3. Abundance Variation of Functional Microbes Related to System Stability and Phosphorus Removal

Sludge was sampled on day 0 (inoculum), day 21 (SRT10d1 and SRT5d), day 84 (SRT10d2 and SRT4d), and day 120 (SRT10d3 and SRT3d) from two operated experimental SBRs for high-throughput sequencing analysis, and the average length of OTU was 416 with a coverage above 99% in this study. The analysis of microbial diversity and community structure was conducted after data extraction flat. The indexes that reflected microbial diversity are shown in Table S3. Microbial diversity reduced compared with the WAS, indicating the reconstruction of microbial communities.

The abundance of functional bacteria related to phosphorus removal and system stability is shown in Table 3 and the details of the bacteria with abundance higher than 1% are shown in Figure S2. The detected sequence numbers of the microbial related to nitrification are shown in Table S4. The key to maintaining the stability of an activated sludge process is to avoid sludge bulking. The enrichment of the filamentous bacteria was one of the most significant factors that caused sludge bulking. The excessive growth of *Thiothrix*, which is a typical filamentous bacterium, results in bulking the sludge in conventional activated sludge systems [21]. *Thiothrix* was detected in the short-sludge-age or ultra-short SRT EBPR systems reported by [13,15]. The abundance of *Thiothrix* was increased to 1.86% in SBR_S-SRT_ after 120 d operation due to the shortening of SRT, but it remained stable and lower than 1% in the SBRcontrol. The disagreement between the enrichment of filamentous bacteria and the good settling ability of sludge in SBR_S-SRT_ was mainly due to the relatively low abundance of *Thiothrix* (ranged as the twelfth most abundant bacteria in the sample SRT3d) and the quality of the influent. However, the results suggest that the SBR_S-SRT_ had the risk of sludge bulking.

Only a few sequences were detected and the sequence numbers of *Nitrospira* and *norank_Nitrosomonadaceae* were both zero in the samples SRT4d and SRT3d, as shown in Table S4. These dates suggest that the nitrifying bacteria were washed out from the SBR_S-SRT_ completely, which is in agreement with the poor ammonia removal performance of SBR_S-SRT_. *Tetrasphaera* is an important PAO [22] and has a comparable contribution to phosphorus removal to *Candidatus_Accumulibacter* in the EBPR process [23]. *Tetrasphaera* was the dominant PAO in both the SBR_S-SRT_ and SBR_control_, while the abundances of *Candidatus_Accumulibacter* and *Dechlorimonas* [24] were lower than 0.5% in the collected samples. The abundance of *Tetrasphaera* in the sample collected from SBRS-SRT at SRT 3 d was almost seven times higher than the inoculum (6.29% vs. 0.81%). The shortening of SRT was beneficial for the enrichment of *Tetrasphaera*. The abundance of *Tetrasphaera* in the samples collected from the SBRcontrol decreased from 12.22% to 3.56% when the duration of the oxic phase extended from 2 h to 2.5 h. The extension of the oxic phase improved the ammonia removal that resulted in the enhanced competition of the carbon source between denitrification and phosphorus release in the anaerobic phase. This might cause the reduction in PAO *Tetrasphaera. Hydrogenophaga* [25,26] and *Flavobacterium* [27] were reported to have the function of phosphorus removal and were enriched in SBR_S-SRT_ and SBRcontrol, respectively. The abundance of the main glycogen accumulating organisms (GAO) *Candidatus_Competibacter* [17] increased from 1.35% to 3.26% when the SRT was shortened from 4 d to 3 d. The wash-out of the nitrifying bacteria in the SBRS-SRT eliminated the competition between denitrification and phosphorus release processes in the anaerobic phase, which left a surplus carbon source that accelerated the enrichment of both GAOs and PAOs. However, the abundance of the PAOs was still significantly higher than the GAOs, which was in line with the excellent phosphorus removal performance of SBR_S-SRT_.

### 3.4. Methanogenic Efficiency of WAS from the Two SBRs

**Figure 4:**
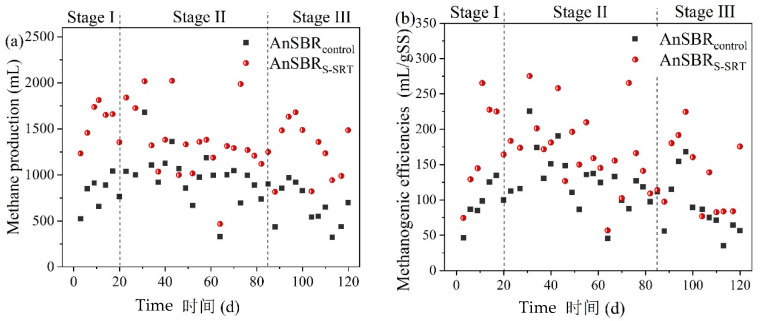
The anaerobic digestion performance of the two experimental AnSBRs (**a**). methane production, (**b**) methanogenic efficiency.

The methane production and methanogenic efficiency of two AnSBRs (AnSBR_control_ and AnSBR_S-SRT_) treated WAS from the corresponding two SBR is demonstrated in Figure 4. The methanogenic efficiency of the AnSBR_S-SRT_ treated WAS was significantly higher than the AnSBR_control_. The WAS average methanogenic efficiency decreased from 166.7 ± 54.7 mL CH_4_/gSS to 136.0 ± 53.2 mL CH_4_/gSS when the SRT of SBR_S-SRT_ shortened from 4 d to 3 d. This result was consistent with the decrease in MLVSS/MLSS ratios (from 60.2% to 54.0%), as previously discussed. However, the average methanogenic efficiency of the WAS collected from the SBR_S-SRT_ (SRT 3 d) was 54.1% higher than the methanogenic efficiency of the WAS collected from the SBR_control_ (the oxic phase duration as 2.5 h), as shown in Table S5. As discussed above, the two SBRS achieved equivalent COD removal efficiency. Taking the increase in *Y_obs_* and comparable COD removal efficiency into an account, the methane recovery potential of SBR_S-SRT_ at SRT 3 d was 95.1% higher than the SBR_control_ with the oxic phase duration as 2.5 h, according to Equations (2) and (3). The S-SRT approach was an effective and promising selection for the enhancement of energy recovery.

## 4. Conclusions

Two EBPR SBR systems were set up to treat real domestic sewage and two AnSBRs were conducted to compare the methanogenic efficiencies of the generated (or collected) WAS from the two systems. A *T*-test was employed to evaluate the significant difference between the two SBRs at each stage. The SBR_S-SRT_ achieved efficient and comparable COD, ${\mathrm{PO}}_{4}^{3-}$-P, and TP removal (*p* > 0.05) and the removal efficiencies were 91.5 ± 3.5%, 95.4 ± 3.8%, and 93.5 ± 3.7%, respectively, at a SRT of 3 d. The SBR_S-SRT_ remained steady sludge settleability, which was comparable with the SBR_control_ (*p* > 0.05). The ammonia in sewage was retained with an ammonia removal efficiency of 15.7 ± 7.7% indicated the wash-out of nitrifiers in SBR_S-SRT_ when the SRT was shorter than T4 d. The enrichment of *Tetrasphaera* and the stable abundance of filamentous bacteria were the source force that maintained the stability of the SBRS-SRT. The methanogenic efficiency increased by 95% when the SRT of SBR_S-SRT_ was 3 d compared with SBR_control_ as its oxic phase duration was 2.5 h. This study proved that the S-SRT EBPR was a promising and practical A-stage selection that maximally utilized the biological phosphorus removal function when shifting a conventional WWTP into a “source and energy recovery factory” operated as an A-B stage process.

## Figures and Tables

**Table 1:** Characteristics of the sewage used in this study.

Characters	COD mg/L	$NH_{4}^{+}$-N mg/L	$PO_{4}^{3-}$-P mg/L	TP mg/L	pH
Ranges	213.3–773.3	39.2–84.9	2.9–6.2	3.7–6.7	7.1–7.5
Average values	477.8 ± 124.4	59.6 ± 7.1	4.1 ± 0.6	5.1 ± 0.8	7.2 ± 0.2

**Table 2:** Operation strategies of the two experimental SBRs.

Reactor	Parameters	Stage I (0–21 d)	Stage II (22–84 d)	Stage III (85–120 d)
SBR_control_	Anaerobic (h)	2.5	2.5	2.0
	Oxic (h)	1.5	1.5	2.0
	SRT (d)	10	10	10
	Aeration (L/min)	0.8	0.8	0.8
SBR_S-SRT_	Anaerobic (h)	2.5	2.5	2.5
	Oxic (h)	1.5	1.5	1.5
	SRT (d)	5	4	3
	Aeration (L/min)	0.8	0.8	0.8

**Table 3:** Functional genera related to phosphorus removal and system stability.

Genera		Inoculum	SRT 10 d1	SRT 10 d2	SRT 10 d3	SRT 5 d	SRT 4 d	SRT 3 d
PAOs	*Tetrasphaera*	0.81	4.28	12.22	3.56	3.43	4.60	6.29
	*Flavobacterium*	0.27	0.45	1.5	1.76	0.54	0.06	0.16
	*Hydrogenophaga*	0.02	0.02	-	0.01	1.65	0.79	1.55
	*Dechloromonas*	0.21	0.22	0.05	0.11	0.02	0.03	0.04
	*Candidatus_* *Accumulibacter*	-	0.01	0.09	0.04	0.06	0.04	0.04
GAOs	*Candidatus_* *Competibacter*	0.42	1.28	4.97	4.23	3.49	1.35	3.26
Filamentous bacteria	*Thiothrix*	0.03	0.04	0.07	0.05	0.08	0.79	1.86

Relative abundance of the functional genera, percent.

## Data Availability

Not applicable.

## Supplementary Materials

The following supporting information can be downloaded at: https://www.mdpi.com/article/10.3390/ijerph19095494/s1. Text S1: Anaerobic sequential batch reactor (AnSBR) set-up and operation, Figure S1: schematic of the two experimental AnSBR, Figure S2: percent of microbial abundance on Genus level (abundance > 1%), Table S1: the average value of the indexes related to the sludge in the two experimental SBRs, Table S2: the average nutrients removal efficiencies of the two experimental SBRs, Table S3: variation of index related to the microbial diversity, Table S4: sequence number of microbial related to the nitrification process without data extrac-tion flat, Table S5: the average methane production and methanogenic efficiencies of the two experi-mental AnSBR.

## Abbreviations

Short sludge retention time	S-SRT	Anaerobic digestion	AD
Sequencing batch reactors	SBR	Mixed liquor volatile suspended solids	MLVSS
Wastewater treatment plants	WWTPs	Mixed liquor suspended solids	MLSS
Enhanced biological phosphorus removal	EBPR	Anaerobic SBR	AnSBR
Anaerobic/anoxic–oxic	AO	Polyethersulfone	PES
Anaerobic–anoxic–oxic	AAO	Methanogenic efficiency	MP
Polyphosphate accumulating organisms	PAOs	Total methane recovery potential	MP_total_
Sludge volume index	SVI	Ammonia oxide bacterial	AOB
Total phosphorus	TP	Glycogen accumulating organisms	GAO
Waste activated sludge	WAS		
