# Supplementary material for: The Feasibility of Maintaining Biological Phosphorus Removal in A-Stage via the Short Sludge Retention Time Approach: System Performance, Functional Genus Abundance, and Methanogenic Potential
Source: Int J Environ Res Public Health. 2022 May 1;19(9):5494. doi: 10.3390/ijerph19095494 (PMC9099736; doi:10.3390/ijerph19095494)
Supplement: Supplementary file 1 [file ijerph-19-05494-s001.zip › ijerph-1588250-supplementary.pdf]

Supplementary information

The feasibility of maintaining biological phosphorus removal in A-stage via short sludge retention time approach: system performance, funcational genuses abundances, and methanogenic potential

Haichao Luo, Chuanming Xing, Wanqian Guo[*], Bo Yan, Qi Zhao, Nanqi Ren

State Key Laboratory of Urban Water Resource and Environment, Harbin Institute of Technology, Harbin 150090, PR China

[*] Corresponding author. Tel./fax: +86 -451-86283008.
Email address: guowanqian@126.com (W.Q. Guo)

Text S1. Anaerobic sequential batch reactor (AnSBR) set-up and operation

Two silk mouth bottle with a working volume of 2 L was used and retrofit into two anaerobic sequencing batch reactor (AnSBR). The cap was provided with two sampling ports for WAS discharge (or input) and biogas collection. The two AnSBR were placed in an air bath shaker. The temperature was set as 37.0 ± 0.1°C and the rotation speed was set as 110 rmp. 300 mL AD WAS was discharged and then 300 mL newly collected WAS was fed into the AnSBR every 3 d to maintain the sludge retention time (SRT) (or hydraulic retention time, HRT) around 20 d.

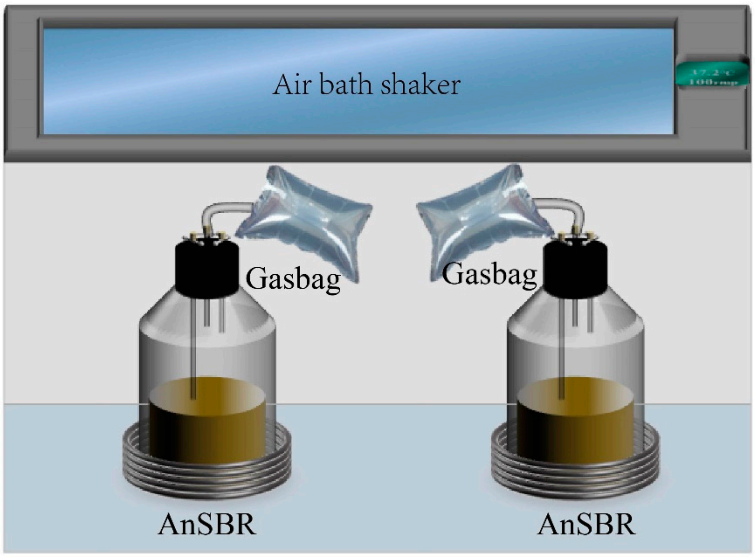

Figure S1. schematic of the two experimental AnSBR

Table S1. the average value of the indexes related to the sludge in the two experimental SBRs

		Stage I		Stage II		Stage III	
		$SBR_{control}$	$SBR_{S\text{-}SRT}$	$SBR_{control}$	$SBR_{S\text{-}SRT}$	$SBR_{control}$	$SBR_{S\text{-}SRT}$
MLSS[1]	Average	3.5	2.0	4.0	1.6	3.4	1.3
	Deviation	0.9	0.3	0.7	0.4	0.5	0.4
	p (t-test)	＜0.05		＜0.05		＜0.05	
MLVSS[1]	Average	2.2	1.4	2.3	1.0	1.7	0.7
	Deviation	0.6	0.2	0.4	0.2	0.3	0.2
	p (t-test)	＜0.05		＜0.05		＜0.05	
SVI[2]	Average	72.5	62.0	49.3	46.6	35.4	46.4
	Deviation	26.4	7.5	14.1	8.5	4.8	9.2
	p (t-test)	0.08		0.20		＜0.05	
MLVSS/ MLSS[3]	Average	63.6	68.9	57.6	60.2	51.2	54
	Deviation	2.2	5.8	3.6	6.1	6.3	8.0
	p (t-test)	＜0.05		＜0.05		0.10	

1. The concentrations of MLSS and MLVSS in the two SBRs, g/L.
2. The sludge SVI of the two SBRs, mL/g.
3. The ratios between MLVSS and MLSS, %.

Table S2. the average nutrients removal efficiencies of the two experimental SBRs

		Stage I		Stage II		Stage III	
		$SBR_{control}$	$SBR_{S\text{-}SRT}$	$SBR_{control}$	$SBR_{S\text{-}SRT}$	$SBR_{control}$	$SBR_{S\text{-}SRT}$
COD[1]	Average	89.9	90.7	91.1	90.7	92.0	91.5
	Deviation	3.9	5.4	3.2	3.5	3.8	3.5
	p (t-test)	0.60		0.50		0.53	
NH_4^+-N[1]	Average	35.2	25.9	66.1	21.8	88.9	15.7
	Deviation	19.9	18.5	13.5	9.2	5.8	7.7
	p (t-test)	0.12		$<$0.05		$<$0.05	
PO_4^{3-}-P[1]	Average	96.9	97.2	96.4	96.1	96.1	95.4
	Deviation	2.3	2.6	4.5	4.3	4.2	3.8
	p (t-test)	0.64		0.68		0.52	
TP[1]	Average	96.2	96.3	94.6	93.8	94.2	93.5
	Deviation	4.0	5.2	4.8	3.9	4.5	3.7
	p (t-test)	0.92		0.29		0.46	

1.The nutrients removal efficiencies，percent.

Table S3. variation of index related to the microbial diversity

	sobs	Shannon	Simpson	Ace	Chao	Coverage
Inoculum	974	4.91	0.0416	1077.35	1078.08	0.9934
SRT10d1	965	5.03	0.0268	1127.45	1112.86	0.9917
SRT10d2	863	5.02	0.0203	1058.55	1082.20	0.9915
SRT10d3	766	4.95	0.0195	915.23	908.29	0.9930
SRT5d	887	4.90	0.0259	1069.18	1073.60	0.9915
SRT4d	729	4.30	0.0479	905.29	873.44	0.9925
SRT3d	804	4.80	0.0216	922.61	912.72	0.9936

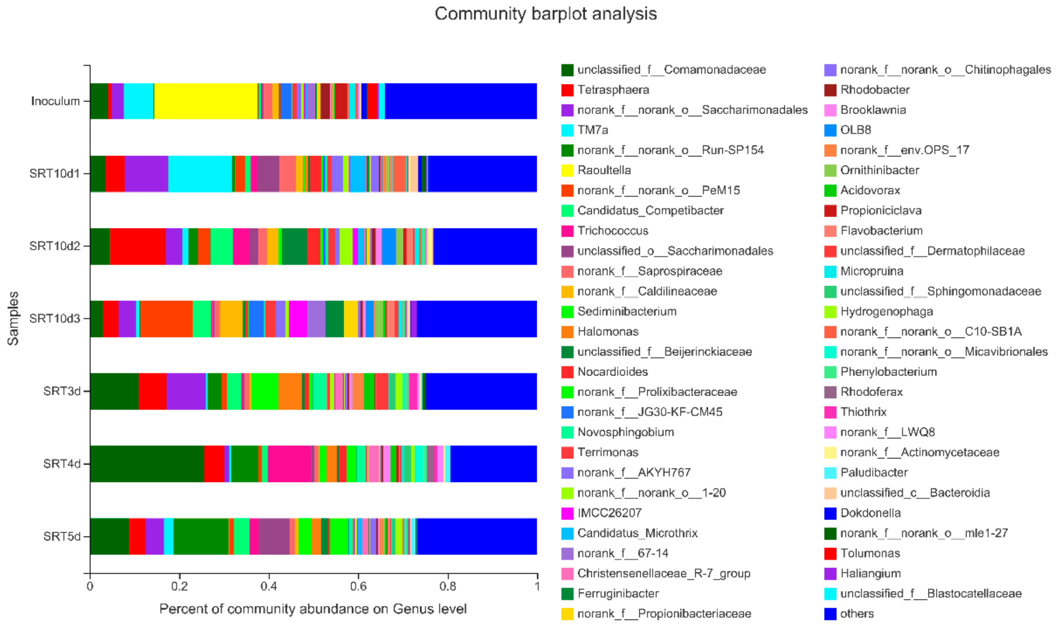

Figure S2. percent of microbial abundance on Genus level (abundance>1%)

Table S4. sequence number of microbial related to the nitrification process without data extraction flat

	WAS	SRT 10d1	SRT 10d2	SRT 10d3	SRT 5d	SRT 4d	SRT 3d
Nitrosomonas	16	19	4	12	1	0	1
Nitrospira	96	13	1	35	0	0	0
norank_ Nitrosomonadaceae	0	1	3	1	2	0	0

Table S5. the average methane production and methanogenic efficiencies of the two experimental AnSBR

		Stage I		Stage II		Stage III	
		AnSBR control	AnSBR S-SRT	AnSBR control	AnSBR S-SRT	AnSBR control	AnSBR S-SRT
Methane production[1]	Average	805.2	1558.2	979.5	1358.3	654.7	1266.3
	Deviation	173.7	213.3	266.7	382.0	217.1	322.5
	p (t-test)	＜0.05		＜0.05		＜0.05	
Methanogenic Efficiency[2]	Average	96.5	175.8	112.5	168.7	88.2	136.0
	Deviation	29.1	66.6	38.4	54.7	41.7	53.2
	p (t-test)	＜0.05		＜0.05		＜0.05	

1. The methane production of the two AnSBR, mL.
2. The methanogenic efficiencies of the two AnSBR, mL/gSS.
